# Measuring trust in one’s physician: A scoping review

**DOI:** 10.1371/journal.pone.0303840

**Published:** 2024-05-17

**Authors:** Linda R. Weber

**Affiliations:** Department of Social and Behavioral Sciences, State University of New York Polytechnic Institute, Utica, New York, United States of America; Faculty of Health Sciences - Universidade da Beira Interior, PORTUGAL

## Abstract

Trust in one’s physician drives positive health practices. However, the conceptualization and subsequent operationalization of trust have become clouded due to the multitude of approaches that have resulted in several different measures with varied dimensions and indicators. The objectives of this scoping review were: 1) to discover any new developments in the measurement of trust, 2) to identify those measures of trust, whether newly created or refined in the last ten years, that have known reliability and validity, and 3) to compare those instruments’ conceptualizations, dimensions, and indicators. This researcher conducted an electronic search of three databases (PubMed, SOCAB, and PsycINFO). Two reviewers screened those selected studies and identified the following six key measurement tools, of which three had shorter, more abbreviated derivatives: the Trust in Physician Scale and its modification, the Wake Forest Physician Trust Scale and its short form, the Health Care Relationship Trust Scale and its refinement, the Trust in Oncologist Scale and its shortened form, the Trust in Health Care Providers Scale, and the Trust in My Doctor Scale. Of these six distinct tools, only the Trust in Oncologist Scale was developed and validated in non-US populations. Also identified were ten dimensions of trust: fidelity, technical competence, communicative competence, interpersonal competence (i.e., caring), honesty, confidentiality, global, behavioral, fairness, and system trust/accountability. Interpersonal competence and fairness emerged as newer dimensions that deserve further study. A comparative analysis of the indicators of these trust dimensions revealed some discrepancies that deserve theoretical and psychometric attention. In addition, incorporating item-response theory to assess measurement invariance has enhanced the assessment of external validity. This review provides a resource for researchers that will lead to a more uniform understanding of trust, thereby setting the basis for future theoretical integration and measurement development.

## Introduction

Trust in one’s physician is a critical force that drives the patient to engage in both preventive and curative health practices that promote health [1–4]. Conceptualized in diverse ways, these approaches to trust tend to center around the seminal work of Anderson and Dedrick [5], who stated: "Interpersonal trust is defined as a person’s belief that the physician’s words and actions are credible and can be relied upon. According to this definition, a patient who trusts his physician believes that his physician will act in his best interest and provide support and assistance concerning treatment and medical care" (p.1092). Supporting this emphasis on a physician who acts in the patient’s best interest, Thom, Hall and Pawlson [6] and Hall, Dugan, Zheng, and Mishra [7] clarify that trust, because of its future orientation and emotional basis, is a different construct than satisfaction with which it is most confused. Trust is also distinct from confidence [8, 9], which has a systems focus detached from risk, and dependence [10], which emerges in situations of heightened risk when the familiarity necessary for trust is absent. These refinements align trust in one’s physician more closely with contemporary understandings of interpersonal trust [11, 12].

Due to theoretical underpinnings yet to be thoroughly integrated [7, 13, 14], an abstract construct such as trust is challenging to measure. In addition, the plethora of measurement tools with various approaches reflect different conceptual understandings that deserve our attention, given the ultimate goal of researchers being more unified in their approach. By untangling these conceptual relationships, we may better understand what trust is, what influences trust, and how trust impacts healthcare service delivery successes. This scoping review hopes to work toward achieving this goal by 1) identifying new developments in the measurement of trust, 2) determining current key measures of trust in the physician with demonstrated reliability and validity with the explicit purpose of 3) comparing trust’s primary dimensions and their operationalization.

This research builds on the 2014 systematic review of Müller, Zill, Dirmaier, Harter, and Scholl [2], who identified primary physician trust measurement tools until 2013. Unlike Müller et al. [2], whose focus was to identify trust measures and their psychometric properties using a systematic review protocol, this study’s primary objective was to add to these identified measures additional measures whose reliability and validity had been assessed from 2013–2024 in order to compare how trust’s dimensions had been conceptualized and operationalized for all primary measurement tools. Munn et al. [15] differentiate systematic and scoping reviews, noting that the former attempts to answer a specific research question, and the latter explores broader questions, such as examining specific factors or dimensions of a concept, which aligns with this study’s comparison of dimensions and indicators. Although this review initially focuses on identifying measurement tools, which appears more systematic, its ultimate objective of identifying and comparing the dimensions and indicators of trust suggests that the scoping protocol is a more appropriate designation.

## Methods

This review identified studies focused on measuring trust in the physician using a scoping review [15, 16] that followed the PRISMA guidelines for scoping reviews [17]; see S1 File. The focal question for this search was: What were the primary research studies that assess the psychometric properties of those tools measuring trust in one’s physician between 2014 and 2023?

### Search strategy

The subsequent identification procedure entailed conducting an online literature search of the most relevant electronic databases, PubMed, SOCAB, and PsycINFO, using some combination of Trust AND Physician-Patient Relationship or Patient or Doctor AND Measurement or Validation or Psychometric, see the S2 File for the detailed search strategy used for each database. This focused approach identified 192 initial articles from PubMed, 22 additional nonduplicates from SOCAB, and eight additional nonduplicates from PsycINFO. These three databases were chosen in discussions with the research librarian as they balanced the need for sensitivity with the need for precision in this topical area [18]; from the author’s experience, significant scholarly works surrounding trust in the physician have appeared in journals included within these databases, a conclusion supported during initial piloting of the database search criteria.

### Study selection

For the screening stage, this researcher trained a student assistant in applying the exclusionary and inclusionary criteria. A trial was done on the first twenty-five articles to assess the commonality of understanding in applying the criteria. Subsequently, this researcher and the student assistant reviewed each of the abstracts and text of the articles (as needed) in tandem, consulting each other as necessary when concerns or questions arose as we applied the inclusionary and exclusionary criteria.

To be included initially, the manuscript must:

1. Be a peer-reviewed journal article,
2. Have been published between June 2013 and July 2023,
3. Have a topical focus on trust,
4. Focus on the trustee as the physician,
5. Address the psychometric measurement of trust in the physician, and
6. Be published in English

Three concerns are noteworthy regarding these inclusionary criteria. First, the search may have missed viable non-English measurement tools due to using the inclusionary criteria of English language publication. Second, this researcher chose the study time frame with the goal of updating Müller, Zill, Dirmaier, Harter, and Scholl’s [2] 2014 review of the quality of existing measures of trust; the search time frame was initiated in 2013 as this was Müller et al.’s endpoint for his search. Finally, even though Müller et al.’s review was a thorough assessment of the quality of the measurement tools, this focus is outside of the scope of this study. Whereas this review will address the psychometric properties of new and existing tools found within our final dataset, the purpose of this review is not to assess the psychometric quality of these tools but to compare the conceptualization and operationalization of tools with known psychometric properties.

The exclusionary criteria applied during the final screening stage were:

1. The focus was not on trust (e.g., confidence, mistrust, and the like),
2. The trustee was not the physician (e.g., nurse, not the health care system, and the like), and
3. The research goal was not to study the psychometric properties of a trust in one’s physician measurement tool, except for systematic reviews or theoretical workups of this topic.

**Fig 1:**
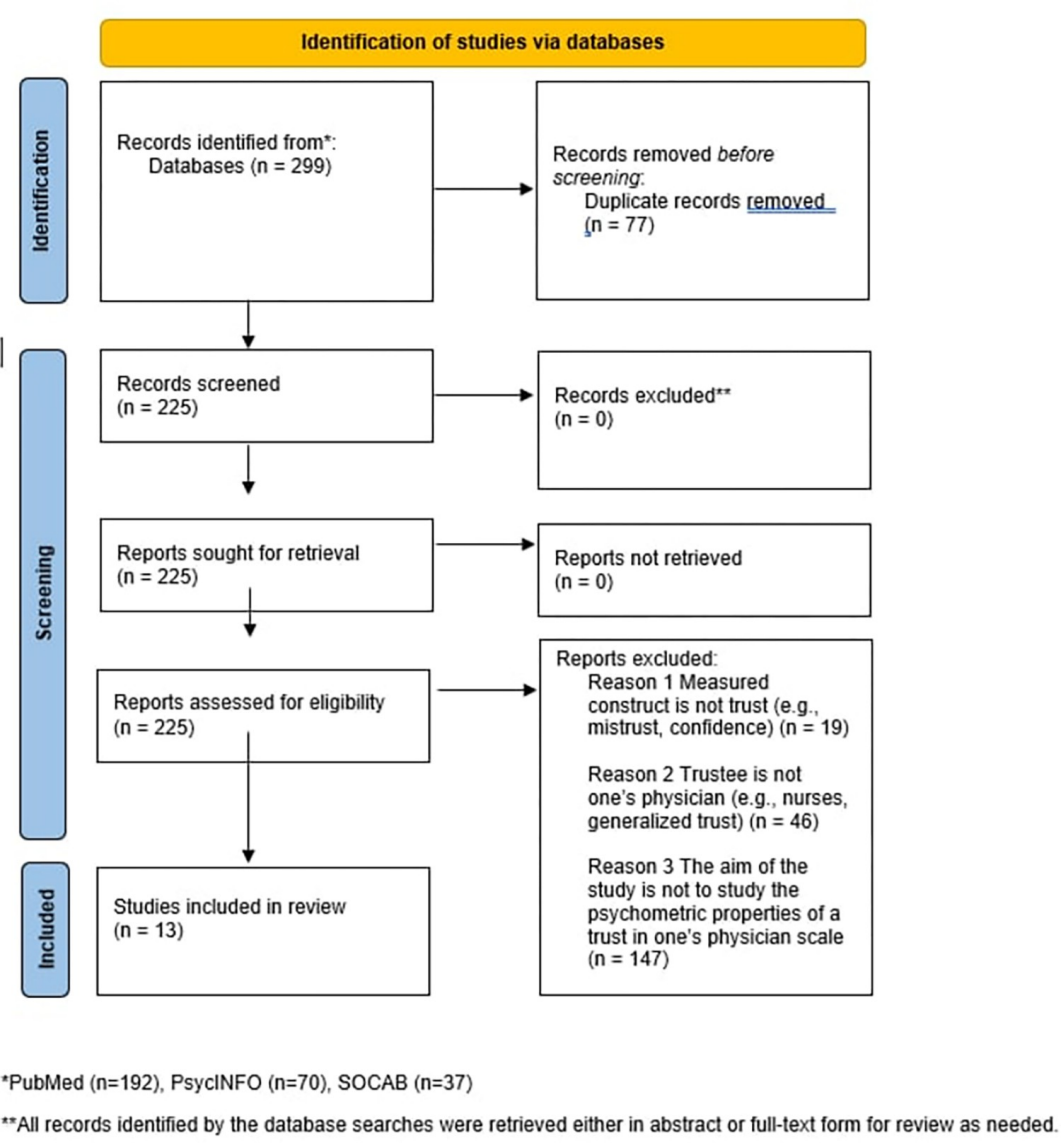
PRISMA flow diagram.

Fig 1 illustrates the overall selection process.

### Data charting methods

Data charting occurred in two stages. The objective of the first charting stage was to identify measurement tools that had undergone assessment for reliability and validity. After the initial selection of all studies for further screening, this researcher and the student assistant identified those tools used to measure trust in the selected studies by a review of the manuscript’s title, abstract, or research methods section to address this first objective. Each of these tools was further charted in terms of the frequency of use within this scoping review database to understand their importance in measuring trust in the physician. The categories for exclusion of the tool from further consideration were: 1)Not Identified = author did not identify measurement tool, 2) Author-Created = author created a tool specifically for their study with little to no psychometric testing as evidenced by the nonexistence of any assessments of construct validity or exploratory or confirmatory factor analysis to confirm the appropriateness of dimensionality, and 3) NA = focus of scale was not physician, the scale was only one part of a larger instrument or little psychometric testing conducted on a non-author created scale. After identifying the tools, subsequent charting involved this researcher reviewing the actual measurement tools in detail to identify the dimensions of trust and their indicators to achieve the second research objective.

In the second stage of the charting process, this researcher reviewed those scholarly studies that survived the inclusionary and exclusionary process to address the objective of identifying new developments in the measurement of trust. The data charted in this step of the process included the following: 1) any new measures created since 2013, 2) any new dimensions in the conceptualization of trust, and 3) any new reliability and validity metrics of existing or new trust measures.

## Results

Three research objectives organized this section: first, to identify critical new developments in the measurement of trust; second, to identify principal measurement tools that had undergone reliability and validity testing; and third, to compare these measures’ dimensions and indicators.

### New developments in the measurement of trust

Applying the inclusionary and exclusionary criteria, this researcher and assistant identified thirteen scholarly articles that comprise the final dataset, representing the current state of the measurement of trust in the physician (see Table 1).

**Table 1:** Descriptive data and psychometric properties of key measurement articles included.

Selected Studies	Scale Used	Sample	Psychometric Properties
1. Aloba O, Mapayi B, Akinsulore S, Ukpong D, Fatoye O. Trust in Physician Scale: factor structure, reliability, validity and correlates of trust in a sample of Nigerian psychiatric outpatients. Asian J Psychiatr. 2014;11:20–7.	Trust in Physician Scale (TiPS)	Nigeria–n = 223 Nigerian psychiatric outpatients	Principle Components Analysis supported a 2-factor solution. **Reliability:** Cronbach’s α = .68. **Validity:** With medication adherence (beta = .268, p = .002), with number of previous admissions (r = .229, p < .001), with schizophrenic relapses (r = .339, p < .001).
2. Bani M, Rossi E, Cortinovis D, Russo S, Gallina F, Hillen MA, et al. Validation of the Italian version of the full and abbreviated Trust in Oncologist Scale. Eur J Cancer Care (Engl). 2021;30(1):e13334.	Trust in Oncologist Scale (TiOS)–Full	Italy–n = 194 Italian oncology patients	4-factor model & 1-factor model- Not confirmed using factor analysis. **Reliability:** Test-Retest (r = .71. p < .001),Internal Consistency Cronbach’s α = .95. **Validity:** With satisfaction (r = .68, p < .001), with willingness to recommend oncologist (r = .66, p < .001).
	TiOS–SF (Short form)	Italy–n = 194 Italian oncology patients	1 Factor Model confirmed using factor analysis. **Reliability:** Test-Retest (r = .51, p < .001), Cronbach’s α = .88. **Construct Validity:** With satisfaction (r = .64, p < .001), with willingness to recommend the oncologist (.67, p < .001).
3. Conradsen S, Lara-Cabrera ML, Skirbekk H. Patients’ knowledge and their trust in surgical doctors. A questionnaire-based study and a theoretical discussion from Norway. Social Theory & Health. 2023;21(1):33–50.	N/A Theoretical Article*		
4. Dong E, Liang Y, Liu W, Du X, Bao Y, Du Z, Ma J. Construction and validation of a preliminary Chinese version of the Wake Forest Physician Trust Scale. Med Sci Monit. 2014;20:1142–50.	Wake Forest Physician Trust Scale -Chinese	China–n = 352 outpatients	Exploratory factor analysis generally supported a 1-factor solution. Confirmatory factor analysis did not confirm a 1-factor model. **Reliability:** Cronbach’s α = .8333. **Validity:** Convergent with patient satisfaction (r = .73. p< .001); Predictive of recommending physician (r = .453, p < .001), occurrence of dispute (r = .209, p < .001), seeking a second opinion (r = .406, p < .001), treatment adherence (r = .444, p < .001), and consideration of switching physicians (r = .471, p < .001) .
5. Gopichandran V, Chetlapalli SK. Trust in the physician-patient relationship in developing healthcare settings: a quantitative exploration. Indian J Med Ethics. 2015;12(3):141–8.	Gopichandran New Scale–Socioculturally Competent Trust in Physician Scale for a Developing Country (SCTPSD)**	n = 625 men and women from rural and urban India	Structural equation modeling using the 12 items indicated a good fit.
6. Gopichandran V, Wouters E, Chetlapalli SK. Development and validation of a socioculturally competent trust in physician scale for a developing country setting. BMJ Open. 2015;5(4):e007305.	SCTPSD	n = 616 rural and urban adults from India	A unidimensional model demonstrated a good fit using confirmatory factor analysis. **Reliability:** Cronbach’s α = .928. **Validity:** Content validity using qualitative inductive interviewing to identify key themes, item-response analysis indicated good characteristic curves and item information for the 12-item scale, with the scale reliable between 2 and +1.
7. Hillen MA, Postma RM, Verdam MG, Smets EM. Development and validation of an abbreviated version of the Trust in Oncologist Scale-the Trust in Oncologist Scale-short form (TiOS-SF). Support Care Cancer. 2017;25(3):855–61.	TiOS -SF	Netherlands–n = 92 Dutch cancer patients	Unidimensional structure confirmed by exploratory factor analysis. **Reliability:** Test-Retest r = .67 (p < .001), Cronbach’s α = .94. **Validity:** Convergent with satisfaction (r_s_ = .524, p < .001) and with trust in the Dutch healthcare system (r_s_ = .289, p < .05); Predictive for recommending the oncologist (r_s_ = .430, p < .001) and contacting the oncologist (r_s_ = .310, p < .01).
8. Kalsingh MJ, Veliah G, Gopichandran V. Psychometric properties of the Trust in Physician Scale in Tamil Nadu, India. J Family Med Prim Care. 2017;6(1):34–8.	Trust in Physician Scale	India–n = 288 patients	Exploratory factor analysis supported a four-factor solution. **Reliability:** Cronbach’s α = .707. **Validity:** Experts found the face and content validity to be acceptable.
9. Merenstein Z, Shuemaker JC, Phillips RL. Measuring Trust in Primary Care. Milbank Q. 2023:40.	N/A Review Article		
10. Müller E, Zill JM, Dirmaier J, Härter M, Scholl I. Assessment of trust in physician: a systematic review of measures. PLoS One. 2014;9(9):e106844-e.	N/A Review Article		
11. Petrocchi S, Labrie NH, Schulz PJ. Measurement invariance of the Short Wake Forest Physician Trust Scale and of the Health Empowerment Scale in German and French women. Journal of Health Psychology. 2020;25(4):558–69.	Wake Forest Physician Trust Scale–Short Form (WFPTS-SF)	n = 217 German-speaking and 217 French-speaking Women	Confirmatory factor analysis demonstrated a good fit for both the German and French participants and supported a one-factor structure. **Reliability:** German participants Cronbach’s α = .86 and inter-rater correlations r_s_ > .46, French participants Cronbach’s α = .86 and inter-rater r_s_ > .55. **Validity:** Partial scalar invariance analysis was accepted, indicating that factor loadings were equivalent across the German and French groups, except for the global measure of trust.
12. Richmond J, Boynton MH, Ozawa S, Muessig KE, Cykert S, Ribisl KM. Development and Validation of the Trust in My Doctor, Trust in Doctors in General, and Trust in the Health Care Team Scales. Social science & medicine. 2022;298:114827.	TiMD	United States–n = 801 Adults	A six-factor solution was identified using exploratory factor analysis and supported using confirmatory factor analysis. **Reliability:** Cronbach’s α = .90. **Validity:** Content validated by six experts, 21 Cognitive interviews with community members; Convergent with Trust in Physician Scale (TiPS) (r = .84, p < .001) and with the Wake Forest Physician Trust Scale (WFPTS) (r = .86, p < .001); Divergent from mistrust (r = -.62, p < .001), and trust in others (r = .17, p < .001) and trust in the federal government (r = .20, p < .001).
13. Taylor LA, Nong P, Platt J. Fifty Years of Trust Research in Health Care: A Synthetic Review. Milbank Q. 2023;101(1):126–78.	N/A Review		

* Even though this study did assess a 3-item measure of trust in an emergency department, it had sparse testing of its psychometric studies. It was included mainly due to its theoretical value.

** Author-created acronym.

Note: r = Pearson’s r, r_s_ = Spearman’s rho

#### Synthesis of new developments in the measurement of trust

A review of these 13 studies revealed several new developments in the measurement of trust, including the development of new tools, the assessment of the generalizability of existing tools, the introduction of new forms of psychometric assessment, and the emergence of new dimensions of trust. First, three new tools have emerged since 2014, including the short form of the Trust in Oncologist Scale (TiOS-SF) [19], The Trust in My Doctor Scale (T-MD) [20], and a Socioculturally Competent Trust in Physician Scale for a Developing Country (SCTPSD) [21]. Second, since 2014, significant progress has been made in testing the generalizability of the Wake Forest Physician Trust Scale (WFPTS), the Trust in Oncologist Scale (TiOS), and the TiPS scales and their modified or abbreviated forms in non-US populations including the Chinese version of the WFPTS, a German and French version of the A-WFPTS [22], the TiPS in a Nigerian population [23], the TiPS in an Asian Indian population [24], and, finally, with Bani, Rossi [25] assessing the validity of the Italian version of the TiOS long and short form. Third, a new and noteworthy advancement in psychometric testing of cross-cultural stability or invariance has been the introduction of item-response theory and testing of measurement invariance [26, 27] of the WFTS-SF by Petrocchi et al. [22] and of the SCTPSD by Gopichandran et al. [21]. Finally, there are several new developments in the conceptualization of trust, including the following: 1) the emergent dimension of inequality as elucidated by Conradsen, Lara-Cabrera and Skirbekk [28] and Gopichandran and colleagues [21, 29, 30], 2) a focus on a specific condition such as oncology with the Trust in Oncologist Scale [TiOS) and its shortened form [19, 31], 3) the inclusion of the dimension of caring [19, 31, 32], and 4) the inclusion of the dimension of social fairness [20].

### The identification of trust in the physician measurement tools

The second objective of this research was to identify trust measurement tools. The initial study selection of 225 scholarly journal articles, see Table 2, yielded several trust in the physician measurement tools within the study period: the Primary Care Assessment Survey (PCAS) [33], the Trust in Health Care Providers Scale (THCPS) [32], the Trust in My Doctor Scale (TiMD) [20], the Health Care Relationship Trust Scale (HCRTS) [34, 35], the Socioculturally Competent Trust in Physician Scale for a Developing Country [30], some version of the Trust in Oncologist Scale (TiOS) [19, 31], the Trust in Physician Scale (TiPS) [5, 6, 36] and some version of the Wake Forest Physician Trust Scale [7, 37].

**Table 2:** Primary tools identified in the search.

Tool	Frequency	Percent
Not Identified	10	4.4
Author-Created	49	21.8
NA	69	30.7
PCAS	2	.9
THCPS	1	.5
TiMD	1	.5
SCTPSD	2	.9
HCRTS	2	.9
TiOS	5	2.0
TiPS	40	17.8
WFPTS	44	19.6
Total	225	100.0

Note: Not Identified = author did not identify measurement tool, Author-Created = author created a tool specifically for their study with little to no psychometric testing, NA = focus of scale was not physician, the scale was only one part of a larger instrument, or little psychometric testing was involved, PCAS = Primary Care Assessment Survey [33], THCPS = Trust in Health Care Providers Scale [32], TiMD = Trust in My Doctor Scale [20], SCTPSD = Socioculturally Competent Trust in Physician Scale for a Developing Country [30], HCRTS = Health Care Relationship Trust Scale [34, 35], TiOS = Some version of the Trust in Oncologist Scale [19, 31], TiPS = Trust in Physician Scale [5, 6, 36], WFPTS = Some version of the Wake Forest Physician Trust Scale [7, 37].

Whereas a review of the whole of this scoping dataset revealed the use of several measures of trust in one’s physician within the study period, the most commonly utilized were: 1) some version of Hall, Zheng [38] the Wake Forest Physician Trust Scale (WFPTS) (19.6%), and 2) some version of Anderson and Dedrick [5] Trust in the Physician Scale (TiPS) (17.8%), see Table 2. Merenstein, Shuemaker and Phillips [39], in their 2023 scoping review of measuring trust in primary care, also identified these two primary measures. This analysis continues by comparing its findings with those tools identified by Müller et al.’s [2] 2014 review, this study’s baseline, and with those tools identified by Merenstein et al.’s [39] 2023 review, see Table 3, to determine the final measurement tools for further conceptualization and operationalization charting and analysis.

**Table 3:** Identified measurement tools by the author.

Measurement Tool	Muller et al. [2014]	Merenstein et al. [2023]	This author
TiPS	√	√	√
TSPPD	√		
WFPTS	√	√	√
A-WFPTS	√	√	√
HCRTS	√	√	√
R-HCRTS	√	√	√
TiOS	√		√
TiOS-SF			√
PCAS		√	
THCPS			√
T-MD			√
SCTPSD			√

PCAS = Primary Care Assessment Survey [33], THCPS = Trust in Health Care Providers Scale [32], TiMD = Trust in My Doctor Scale [20], SCTPSD = Socioculturally Competent Trust in Physician Scale for a Developing Country [30], HCRTS = Health Care Relationship Trust Scale [34, 35], TiOS = Some version of the Trust in Oncologist Scale [19, 31], TiPS = Trust in Physician Scale [5, 6, 36], WFPTS = Some version of the Wake Forest Physician Trust Scale [7, 37].

To compare dimensions and indicators, this researcher identified those measurement tools that had undergone validity and reliability testing. Like Müller et al. [2] and unlike Merenstein et al. [39], this study excluded the Primary Care Assessment Survey (PCAS) [33] from further consideration as the measurement of trust in one’s physician was only one component of a much larger instrument. In addition, this analysis did not consider further the Trust Scale for the Physician-Patient Dyad (TSPPD) [40] identified by Müller et al. [2] due to the paucity of evidence of further psychometric testing and the limited use of this scale in scholarly research, findings verified with a secondary Google Scholar citation search. Finally, this researcher excluded the SCTPSD [21, 30] from further review because it is a new tool lacking comprehensive psychometrics assessment and investigation outside India.

In sum, the six measurement tools and their derivatives (Table 3) that will provide the focus of this analysis are:

- The Trust in Physician Scale (TiPS) and its modification [5, 6, 36],
- The Wake Forest Physician Trust Scale (WFPTS) [38] and its short form [37],
- The Health Care Relationship Trust Scale (HCRTS) and its refinement [34, 35],
- The Trust in Oncologist Scale (TiOS) [31] and its shortened form [19],
- A Trust in Health Care Providers Scale (THCPS) [32], and
- The Trust in My Doctor (T-MD) Scale [20].

**Fig 2:**
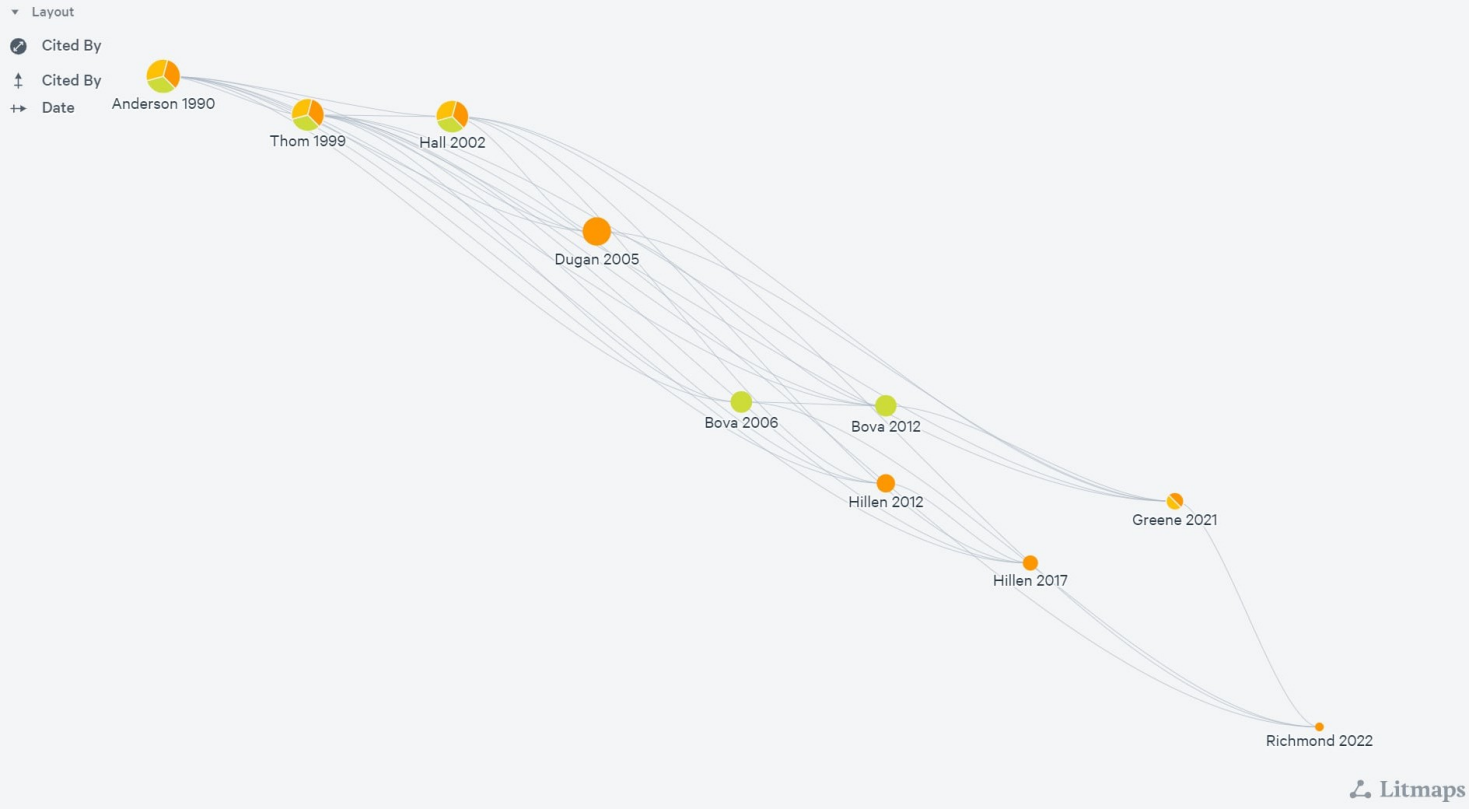
Citational relationships map of identified measurement tools.

These six scales have considerable testing of their psychometric properties (see Table 1). A detailed review of the psychometric quality of these tools is outside this study’s scope; however, see Müller et al. [2] and Merenstein et al. [39] or the instrument authors’ original works, cited above, for more detail. In sum, although psychometric testing of the dominant tools has grown since 2014, much more work still needs to be done. To visualize the citational relationship of these tools, see Fig 2.

This analysis proceeded by charting the dimensions of trust from studies within the included dataset or the measurement tools’ studies of origin, see Table 4, and illustrated the trust indicators in Table 5.

**Table 4:** Primary measures of trust in the physician.

Dimension	Trust in Physician Scale (TiPS)^a^	Wake Forest Physician Trust Scale (WFPTS)^a^	Health Care Relationship Trust Scale (HCRTS)	Trust in Oncologist Scale (TiOS)	Trust in Health Care Providers Scale (THCPS)	Trust in My Doctor (T-MD)
Fidelity	√	√	√	√		√
Competence • Technical • Communication • Interpersonal/Care	√	√	√ √	√ √	√ √ √	√
Honesty	√	√		√		
Confidentiality	√					√
Global		√		√		√
Behavior	√					
Fairness						√
System Trust/Accountability						√

Notes: √ = dimension included in trust scale

^a^ The TiPS and the WFPTS are unidimensional. For these two scales, this researcher determined which dimension or factor with which the indicator was most compatible after examining the other scales.

**Table 5:** Operationalizing trust in one’s physician.

SCALE	TRUST IN PHYSICIAN SCALE (TIPS)	WAKE FOREST PHYSICIAN TRUST SCALE (WFPTS)	HEALTH CARE RELATIONSHIP TRUST SCALE (HCRTS)	TRUST IN ONCOLOGIST SCALE (TIOS)	TRUST IN HEALTH CARE PROVIDERS SCALE (THCPS)	TRUST IN MY DOCTOR (T-MD)
**Dimensions**	Unidimensional^a^	Unidimensional^a^	Interpersonal Connection, Respectful Communication, Professional Partnering	Competence, Fidelity, Honesty, Global, and Caring	Communication, Caring, Competence	Communication Competency, Fidelity, Systems trust, Confidentiality, Fairness, and Global trust
**Fidelity**						
A. Physicians’ interests						My doctor puts making money above my needs
						My doctor recommends expensive treatments to make money
						My doctor might experiment on me without my knowledge
						My doctor rushes through appointments
		Sometimes your doctor cares more about what is convenient for him or her than about your medical needs^b^				
	My doctor is usually considerate of my needs and puts them first	Your doctor only thinks about what is best for you		Your doctor only thinks about what is best for you		
	I trust my doctor to put my medical needs above all other considerations when treating my medical problems					
B. Dr. Agency	I feel my doctor does not do everything he/she should about my medical care	Your doctor will do whatever it takes to get you all the care you need	My HCP is committed to providing the best care possible (Professional Partnering)	Your doctor will do whatever it takes to get you all the care you need^c^		
C. Patient Agency/Professional Partnering				Your doctor always tells you everything you want to know about your illness		
				Your doctor explains everything so you can consent to medical treatment		
				Your doctor strongly cares about your health		
			How often does your HCP discuss options and choices with you before health care decisions are made? (Professional Partnering)			
			I feel that other patients get better care from their HCPs^d^ (Professional Partnering)			
			I feel comfortable talking to my HCP about my personal issues (Professional Partnering)			
			I feel better after seeing my HCP (Professional Partnering)			
			How often do you think about changing to a new HCP(Professional Partnering)			
**Competence -Technical**	TiPS	WFPTS	HCRTS	TiOS	THCPS	T-MD
	My doctor is a real expert in taking care of medical problems like mine// (Thom modification: My doctor is well qualified to manage (diagnose and treat or make appropriate referral) medical problems like mine)	Your doctor’s skills are not as good as they should be				My doctor knows how to treat my medical problems
	I trust my doctor’s judgments about my medical care	You completely trust your doctor’s decisions about which medical treatments are best for you.		Sometimes you worry that your doctor’s medical decisions are wrong		
	I sometimes distrust my doctor’s opinions and would like a second one					
		Your doctor is extremely thorough and careful^b^		Your doctor is very careful and precise	My doctor is thorough and careful	
						My doctor follows up when needed
					I have confidence in the medical care provided by my doctor/provider	
				You think your doctor can handle any medical situation, even a very serious one^c^		
**Competence -Communication**	TiPS	WFPTS	HCRTS	TiOS	THCPS	T-MD
			How often does your HCP talk over your head^d^		My doctor explains things in a way that is easy for me to understand	My doctor explains the benefits and risks of treatment to me
		Sometimes your doctor does not pay full attention to what you are trying to tell him or her	My HCP is an excellent listener	Sometimes your doctor does not pay full attention to what you are trying to tell him/her (fidelity)	My doctor listens carefully to what I have to say	My doctor listens to me
						My doctor believes me when I say something is wrong
					My doctor tells the truth even if it is bad news	
			My HCP makes me feel that I am worthy of his/her time and effort			
			How often does your HCP consider your need for privacy			
**Competence–Interpersonal/Care**	TiPS	WFPTS	HCRTS	TiOS	THCPS	T-MD
	I doubt that my doctor really cares about me as a person			You have doubts about whether your doctor really cares about you as a person	My doctor cares about me as a person	
				My doctor listens with care and concern to all the problems you have^c^		
				Your doctor is available for you whenever you need him/her		
					My doctor takes my questions and concerns seriously (Caring)	
				Your doctor always takes his/her time with you	My doctor does not spend enough time with me (Caring)	
			My HCP is sincerely interested in me as a person			
			My HCP accepts me for who I am			
			My HCP tells me the complete truth about my health-related problems			
			My HCP treats me as an individual			
			My HCP takes the time to listen to me during each appointment			
**Honesty**	TiPS	WFPTS	HCRTS	TiOS	THCPS	T-MD
	I trust my doctor to tell me if a mistake was made about my treatment					
	If my doctor tells me that something is so, then it must be true					
		Your doctor is totally honest in telling you about all of the different treatment options available for your condition^b^		Your doctor is totally honest in telling you about all of the different treatment options available for your condition^c^		
				My doctor would always tell me the truth about my health, even if there was bad news		
				Your doctor always gives you honest information about your prospects		
**Confidentiality**	TiPS	WFPTS	HCRTS	TiOS	THCPS	T-MD
	I sometimes worry that my doctor may not keep the information we discuss totally private					My doctor keeps my medical records private
						My doctor uses secure systems to store medical records
						My doctor respects my privacy
						
						
						
**Global**	TiPS	WFPTS	HCRTS	TiOS	THCPS	T-MD
		All in all, you have complete trust in your doctor^b^		All in all, you have complete trust in your doctor^c^		All things considered, I trust my doctor
						I put my trust in my doctor
						My doctor is trustworthy
				You have no worries about putting your life in [your doctor’s] hands		
**Behavioral**	TiPS	WFPTS	HCRTS	TiOS	THCPS	T-MD
	I trust my doctor so much I always try to follow his/her advice					
**Fairness**	TiPS	WFPTS	HCRTS	TiOS	THCPS	T-MD
						My doctor would treat me fairly, regardless of my ability to pay
						My doctor would treat me fairly, regardless of my race or ethnicity
						My doctor would treat me fairly, regardless of my gender (e.g., male, female, nonbinary)
						My doctor would treat me fairly, regardless of my sexual orientation (e.g., straight, gay, lesbian, or bisexual)
						My doctor would treat me fairly, regardless of my weight
						My doctor would treat me fairly, regardless of my religion
						My doctor would treat me fairly, regardless of my education level
Systems Trust/Accountability	TiPS	WFPTS	HCRTS	TiOS	THCPS	T-MD
						My doctor would be held accountable if they made a mistake
						My doctor would be held accountable if they treated me unfairly
						MY DOCTOR WOULD BE HELD ACCOUNTABLE IF THEY DISCRIMINATED AGAINST ME

Notes: ^a^-The TiPS and the WFPTS are unidimensional. This author reviewed the indicators to determine what dimension or factor it was most compatible to determine table placement after examining the other scales

b–Item included in abbreviated Wake Forest Trust in Physician Index.

^c-^Item included in the abbreviated TiOS

^d^-removed from the refined version of the HCRTS

### Synthesis of the dimensions and indicators of trust

Ten dimensions of trust emerged from the review of the six measurement approaches identified, each with a variety of different indicators. These dimensions are fidelity, competence/technical, competence/communication, competence/interpersonal, honesty, confidentiality, global trust, behavioral, fairness, and systems accountability. This section proceeds with an overview of the conceptualization of the ten dimensions of trust and their operationalization, with attention given to any inconsistencies.

#### Fidelity

Fidelity is the core dimension of trust found in five of the six measures. Mechanic and Meyer [14] define fidelity as having an agency and fiduciary component that involves a "commitment to the patient and patient advocacy" as evidenced by "advocates, argues for, defends, does everything in their power, in my best interests, fights for, doesn’t give up, never stops trying, on my side, sticks up for you, puts self on the line" (p. 663). The analysis of the measurement tools reveals three sub-dimensions of fidelity, each with a different focus: physician’s interests, physician agency, and patient agency.

Fidelity requires the physician to act in the patient’s best interest [6], with the physician’s self-interest of lesser importance [14, 20, 32]. "Your doctor only thinks about what is best for you," as found in the WFPTS [38] and the TiOS long and short forms [19, 31], are representative indicators of fidelity. More recently, the TiMD scale operationalized fidelity by focusing on physician interests with indicators such as "My doctor puts making money above my needs" [20]. Also operationalized as agency, fidelity as physician agency includes indicators such as "Your doctor will do whatever it takes to get you all the care you need," as found in the TiOS [19, 31]. Patient agency, also referred to as "professional partnering" [34, 35], reflects those actions of the physician that empower the patient to take care of their health with such indicators as "Your doctor explains everything so you can consent to medical treatment," as found in the TiOS [19, 31]. In summary, including physician agency as a form of fidelity is essential in measuring trust; further study should investigate whether including a physician’s specific interests is necessary and how patient agency serves as a form of fidelity in creating trust.

#### Competence -technical

As an essential dimension of trust, competence includes a technical component, namely having the skills (e.g., education, credentials, and expertise) necessary to provide good medical care [14]. Whereas most patients do not know the physician’s actual level of expertise [14], they do make such judgments, as directly indicated by "My doctor knows how to treat my medical problems," as found in the TiMD scale [20], and indirectly indicated by "Your doctor is extremely thorough and careful," in the WFPTS [37, 38]. In summary, assessing technical competence appears critical for evaluating patient trust, as it is present in all six scales except for the HCRTS [34, 35].

#### Competence–communication

The distinction between being competent and how one communicates one’s competence warrants its inclusion as a separate dimension of trust. Interpersonal skills [14, 38] are a type of communication competence [20] that includes listening, providing detailed explanations, and speaking in a fashion that is clear for those with no medical background [32]. Communicative competence includes indicators such as "My doctor explains things in a way that is easy for me to understand" and "My doctor listens carefully to what I have to say," as found in the THCPS [32]. In summary, the interrelationships between technical and communication competence as separate sub-dimensions still need further theoretical and research clarification. For example, the relationship between the patient’s perception that the doctor listens carefully (communication competence) and the doctor being thorough and careful (technical competence) needs disentanglement.

#### Competence—interpersonal/caring

The importance of caring is a newer initiative in the understanding of trust, although as far back as 2000, Mechanic [14] asserted that "caring, concern, and compassion… was the most common aspect of trust reported" (p.662). Mercer and Maxwell [41] developed caring in their consultation and relational empathy (CARE) instrument. Within the framework for this study, caring is a form of interpersonal competence of the physician. Hillen et al. [19] identified caring as a distinct dimension of trust and defined it as "the oncologists’ involvement, sympathy, and devotion of attention to the patient" (p.856). The THCPS [32] further developed this construct with indicators such as "My doctor cares about me as a person." Logically, the patient assumes that the doctor who cares about them as an individual will act in their self-interest; this may be why some of the indicators of "caring" also emerged as indicators of fidelity. For example, "My doctor rushes through appointments" in the TiMD [20] and "My doctor does not spend enough time with me" in the THCPS [32] both refer to the amount of time; however, the former uses time as an indicator of fidelity and the latter as an indicator of caring. In summary, for the patient to perceive that the doctor is caring requires the doctor to be aware of their actions and how the patient understands them and, as such, is conceptualized as a form of competence, implying that it is a skill that one can and should learn. Caring appears to be emerging as a critical dimension of trust that challenges the more distant and objective form of the doctor-patient relationship that characterizes Western medicine if one believes trust is essential in delivering quality medical care.

#### Honesty

Honesty refers to telling the truth and avoiding misleading the patient concerning their medical condition, prognosis, and medical care [38], reflecting the level of integrity in the physician-patient relationship [20]. Whereas honesty in some form is part of three primary measures of trust, more recently, the THCPS of Greene and Ramos [32] and the T-MD of Richmond, Boynton [20] did not include this dimension. Interestingly, Bova et al. [35] in the HCRTS included "My HCP tells me the complete truth about my health-related problems" as a measure of caring or interpersonal competence. In summary, the relationship between truth-telling as a marker of the physician’s honesty and how that relates to caring and fidelity deserves further theoretical and empirical examination.

#### Confidentiality

Confidentiality refers primarily to properly using patient information and maintaining its privacy [20, 38]. Confidentiality is one of the least developed dimensions, with Mechanic and Meyer [14], Thom, Hall and Pawlson [6], Hillen, Koning [31], and Greene and Ramos [32] not supporting it as a dimension of trust. The TiPS includes it as a measure of trust with the indicator, "I sometimes worry that my doctor may not keep the information we discuss totally private" [5]. The T-MD has the most developed dimension of confidentiality with indicators such as "My doctor uses secure systems to store medical records" [20]. Theoretically, patient confidentiality may be more of a systemic concern rather than an interpersonal issue [8–10]. In summary, the role of confidentiality in physician trust deserves future investigation psychometrically and theoretically before including it as a dimension of trust. Healthcare systems should promote their efforts to maintain the privacy of patient information even if it is not a dimension of trust, as it sets the grounds for confidence in a system that is necessary to build trust interpersonally.

#### Global trust

As a summative measure of trust, Hall and Zheng [38] describe this dimension as the "irreducible soul of trust" (p. 298). "All things considered, I trust my doctor," as found in the T-MD [20], is one such indicator of global trust. Another indicator provides a contrast to this dimension’s problematic operationalization of "You have no worries about putting your life in [your doctor’s] hands," as found in the WFPTS [38]. This dimension of trust, like confidentiality, has little empirical support for its inclusion. Three of the six trust measures did not include a global measure, with Richmond et al. [20] having the most developed dimension with three indicators. In summary, future research should explore the role of a global measure of trust within a trust index; perhaps its role may be best in testing convergent validity.

#### Behavioral

Certain behaviors of the patient indicate their trust [5]. The TiPS is the only measurement tool with a behavioral indicator of trust: "I trust my doctor so much I always try to follow his/her advice" [5, 6, 42]. This dimension deserves further scrutiny because of its absence in any of the other measurement tools in this analysis, and, as such, there is little empirical support for its inclusion in a measure of trust. In summary, differentiating the construct of trust from the behaviors it promotes needs attention in further developing this indicator.

#### Fairness

Fair or equitable treatment is an important newer initiative; according to Richmond et al. [20], fairness involves the perception of disadvantaged patients that their physicians are equitably treating them. Indicators of fairness include the belief that "My doctor would treat me fairly regardless of my ability to pay/race or ethnicity," as found in the T-MD [20]. Gopichandran and Chetlapalli [30] also address perceived stigma or discrimination in their study of the physician-patient relationship in an underdeveloped nation. In summary, fairness needs to be further studied to understand its role in systems of inequality and how that influences trust.

#### System’s trust/accountability

Richmond and Boynton [20] define systems trust as "belief in institutions, processes, and policies of the health system" (p.2). Systems trust, a belief that the health care system will hold its workers accountable, sets the grounds for interpersonal trust [11] and is indicated by such statements as "My doctor would be held accountable if they made a mistake," as found in the T-MD [20]. The idea of a more institutionally grounded social trust and a more individually oriented interpersonal trust is a distinction made by medical researchers such as Mechanic [13]. Whereas systems trust and interpersonal trust have long been treated as separate domains within the study of trust, understanding the interface between systems trust, confidentiality, and interpersonal trust deserves further attention in this research arena.

## Discussion: Measuring trust

This scoping review initially selected 225 scholarly articles from which further screening resulted in the inclusion of 13 manuscripts in the final dataset that represented research since Mueller et al.’s [2] 2014 review of the quality of psychometric tools measuring trust in the physician; assessment of these manuscripts to identify new developments in the measurement of trust resulted in achievement of this review’s first objective. Identification of six primary trust measurement tools (and their abbreviated forms) that had undergone considerable reliability and validity testing achieved this review’s second objective. Combining these tools with those identified in Mueller et al.’s [2] resulted in the study dataset. Using this dataset, charting the ten dimensions of trust and their operationalization enabled a comparison, which is this review’s third objective.

Regarding the first objective, three noteworthy developments have occurred since Mueller et al.’s [2] 2014 systematic review. Altogether, these developments indicate that the understanding of trust in the physician is still of concern as it is associated with positive healthcare outcomes [1]. The first significant development in measurement was the emergence of two new scales: the short form of the Trust in Oncologist Scale-Short Form (TiOS-SF) [19] and the Trust in My Doctor Scale (T-MD) [20]. The importance of TiOS-SF is not so much in its brevity but in the continual emphasis that medical context influences trust. Whereas short forms are not crucial within a research context, assessing trust using a short form is practically important in the clinical setting, so many of the most often used tools measuring trust have an associated short form.

The second significant development was the emergence of two new dimensions of trust: interpersonal competence/caring and fairness. The continued need for tools to address trust in the context of specific health conditions and power imbalances connects the first and second new developments. Concerning the former, Hillen et al. [19, 43] evidenced that trust was crucial for oncology patients due to the life-or-death nature of their condition; certain factors such as fidelity, competence, honesty, and caring were critical, whereas confidentiality was not. Concerning the latter, Richmond et al.’s TiMD [20] brings to the forefront power imbalances within the physician-patient relationship and how perceptions of fairness are a critical element of trusting relationships. The dimensions of caring and fairness deserve more research and theoretical attention to discern their role in trusting professional relationships. In the past, the medical trust literature neglected these two dimensions, but they are critical for establishing interpersonal trust [11].

The final new development is the external validation of existing and newly created tools aided by item-response theory and the testing of measurement invariance. Trust may indeed be a "variform universal" [44] or "form of association" [12, 45] that transcends cultures in form but is culturally specific in content. Determining which is the case is critically important when assessing trust in a culturally sensitive manner [46]. As researchers assess the validity of a measurement tool, they need to carefully evaluate the cultural context in which it is utilized and determine the meaning of trust within a community or diverse sub-population.

The identification of six predominant measurement tools and their derivatives addressed the second objective. These tools are as follows: the Trust in Physician Scale (TiPS] and its modification [5, 6, 36], the Wake Forest Physician Trust Scale (WFPTS) [38] and its short form [37], the Health Care Relationship Trust Scale (HCRTS) and its refinement [34, 35], the Trust in Oncologist Scale (TiOS) [31] and its shortened form [19], the Trust in Health Care Providers Scale (THCPS) [32], and the Trust in My Doctor (T-MD) Scale [20]. An examination of these tools allowed the realization of the third objective of this research, the identification of ten dimensions of trust, each with a variety of different indicators: fidelity, competence/technical, competence/communication, competence/interpersonal, honesty, confidentiality, global trust, behavioral, fairness, and systems accountability. The analysis of the dimensions and indicators of trust revealed that confidentiality, global trust, behavioral, and system trust have little empirical and theoretical support for their inclusion in a multidimensional measure of physician and patient trust.

In sum, researchers approach the measurement of trust with various understandings and methods that are sometimes contradictory. For the study of trust to move forward, a theoretically integrated model of trust in the physician must be built upon these research findings to date so that the dimensions and the operationalization of trust may be more cohesive. Such a model will provide insight into the practice of trust in the clinic. For example, suppose a patient’s perception that the doctor cares about them as a person is a critical element of trust. In that case, trainers can educate clinicians about the importance of demonstrating care and how to do so. This scoping review provides an in-depth understanding of the state of the art in measuring trust, a first step in achieving this larger goal of creating trusting relationships between physician and patient.

For future practitioners and researchers, a measurement model that differentiates between the causes or determinants of trust, its underlying factors, and its behavioral outcomes is critical for developing a cohesive body of knowledge that aids in the development of doctor-patient relational interventions; such an advancement would support Gopichandran and Chetlapalli’s [30] assertion of the importance of such distinctions in their work in developing healthcare settings.

### Strengths and limitations

The main strength of this study is its focus on comparing the conceptualization and operationalization of the primary tools in the measurement of trust in the physician. Whereas Mueller et al.’s [2] 2014 systemic review of the psychometric quality of the tools to date delved into their reliability and validity, the dimensions of trust and a comparison of their indicators was not one of this study’s goals. In addition, whereas Merenstein et al.’s [39] 2023 review did list the indicators, its primary focus was identifying existing reliable and valid scales of physician trust, and they did not do a comparative assessment of trust’s dimensions and indicators.

Another strength of this study is its use of a detailed and comprehensive electronic database search initially to identify those scholarly articles focused on the psychometric analysis of existing scales or new scales since 2014. In addition, two reviewers working in tandem screened all studies identified in the initial selection. Through this intensive search process, this study identified studies that provide a solid foundation for researchers to develop and refine trust in one’s physician conceptually and operationally.

The primary limitations of this focused review include its focused approach, which may have missed relevant studies. In addition, the use of two non-independent reviewers during the initial screening process may have biased the selection. However, identifying measurement tools and whether the study involved reliability and validity testing as a focus was relatively straightforward.

Another limitation is that this scoping review focuses only on articles published in English; as such, the search protocol may have missed some critical publications. More importantly, the dominance of measures developed in the US may have been an artifact of the English publication inclusion criteria. For this reason, those studying trust in non-US populations should exert care when applying these findings. If this is the case, measurement tools developed in more Euro-centric populations must be carefully validated when used in other populations, as does recent research on validating these tools in non-English and non-US populations.

## Conclusion

In conclusion, this scoping review provided insight into the status of trust-in-physician measurement tools. Six tools with known reliability and validity dominate the field of physician-patient trust research: the TiPS, the AFPTS, the HCRTS, the TiOS, the THCPS, and the T-MD scale. Examining these tools led to identifying ten dimensions of trust: fidelity, competence/technical, competence/communication, competence/interpersonal, honesty, confidentiality, global trust, behavioral, and fairness. Of these, the inclusion of confidentiality, a global measure of trust, and a behavioral dimension deserve scrutiny before inclusion in future measures. The emergence of communication competence, interpersonal competence as caring, and fairness are important new developments in the conceptualization and operationalization of trust. In addition, more recent use of the assessment of measurement invariance using item response adds to the ability to generalize these tools for use in different populations.

Whereas the construction of a valid and reliable measure of trust in one’s physician has progressed in the last ten years, researchers should give attention to measurement concerns that arise from the conceptual and theoretical integration of trust’s dimensions to arrive at a unified and complete understanding of this critical dynamic within the doctor-patient relationship. If one examines interpersonal trust sociologically and holistically as a form of association shaped by the social system and inequality structure of which it is part [11], one can develop a complete understanding of trust as a crucial social force that undergirds social relationships such as that between physician and patient.

## Supporting information

S1 File Prisma checklist. (DOCX)

S2 File Detailed search procedures. (DOCX)
