# Supplementary material for: Measuring trust in one’s physician: A scoping review
Source: PLoS One. 2024 May 17;19(5):e0303840. doi: 10.1371/journal.pone.0303840 (PMC11101118; doi:10.1371/journal.pone.0303840)
Supplement: S2 File — (DOCX) [file pone.0303840.s002.docx]

# S2 File. Detailed search procedures

Search Dates: June 21, 2013 – June 24, 2023

Final PubMed Search 6/24/2023

192 results

((trust*[Title]) AND (physician*[Title] OR doctor*[Title] OR surgeon*[Title] OR clinician*[Title] OR oncologist*[Title])) AND (valid*[Title/Abstract] OR measure*[Title/Abstract] OR psychometric*[Title/Abstract] OR metric*[Title/Abstract] OR scale*[Title/Abstract] OR quantitative[Title/Abstract] OR questionnaire*[Title/Abstract] OR survey*[Title/Abstract]) AND ((y_10[Filter]) AND (english[Filter])) AND ((y_10[Filter]) AND (english[Filter]))

Final PsycINFO

Limiters: Published Date: 20130601-20230731; Publication Type: Peer Reviewed Journal; English; Population Group: Human

70 Results

TI Trust AND AB ( Physician* OR doctor* OR surgeon* OR clinician* OR Oncologist* ) AND AB ( Valid* OR Measure* OR Psychometric* OR metric* OR Scale* OR Quantitative* OR Questionnaire* OR Survey* )

http://sunypoly.idm.oclc.org/login?url=https://search.ebscohost.com/login.aspx?direct=true&db=psyh&bquery=TI+Trust+AND+AB+(+Physician*+OR+doctor*+OR+surgeon*+OR+clinician*+OR+Oncologist*+)+AND+AB+(+Valid*+OR+Measure*+OR+Psychometric*+OR+metric*+OR+Scale*+OR+Quantitative*+OR+Questionnaire*+OR+Survey*+)&cli0=DT1&clv0=201306-202307&cli1=PT11&clv1=0110&cli2=LA1&clv2=Y&cli3=MR1&clv3=18*&cli4=DX1&clv4=Y&type=1&searchMode=Standard&site=ehost-live&ssl=y

Final SOCAB Search July 2, 2023

37 results

Searched for:title(Trust) AND noft((Physician* OR doctor* OR surgeon* OR clinician* OR Oncologist*)) AND noft(Valid* OR Measure* OR Psychometric* OR metric* OR Scale* OR Quantitative* OR Questionnaire* OR Survey*) AND stype.exact("Scholarly Journals") AND la.exact("English") AND PEER(yes) AND pd(20130621-20230624)

Limited by: Date: From 21 June 2013 to 24 June 2023, Source type: Scholarly Journals, Language:English Databases: Sociological Abstracts
